# Distinct High-Profile Methylated Genes in Colorectal Cancer

**DOI:** 10.1371/journal.pone.0007012

**Published:** 2009-09-11

**Authors:** Pooneh Mokarram, Krishan Kumar, Hassan Brim, Fakhraddin Naghibalhossaini, Mehdi Saberi-firoozi, Mehdi Nouraie, Robert Green, Ed Lee, Duane T. Smoot, Hassan Ashktorab

**Affiliations:** 1 Department of Internal Medicine and Gastroenterology Research Center, Shiraz, Iran University of Medical Sciences, Tehran, Iran; 2 Department of Medicine and Cancer Center, Howard University, College of Medicine, Washington, D. C., United States of America; 3 Department of Pathology, Howard University, College of Medicine, Washington, D. C., United States of America; Dr. Margarete Fischer-Bosch Institute of Clinical Pharmacology, Germany

## Abstract

**Background:**

Mutations and promoters' methylation of a set of candidate cancer genes (CAN genes) are associated with progression of colorectal cancer (CRC). We hypothesized that these genes' promoters are inactivated through epigenetic silencing and may show a different profile in high-risk populations. We investigated the status of CAN gene methylation and CHD5 protein expression in African American CRC tissue microarrays (TMA) using immunohistochemical staining.

**Methodology/Principal Findings:**

The promoter methylation status of the CAN genes was studied by methylation-specific PCR (MSP) in 51 Iranians (a white population) and 51 African Americans (AA). Microsatellite instability (MSI) was analyzed as well. The differential frequency of methylation for each gene was tested by chi-square analysis between the two groups based on matched age and sex. CHD5 protein expression was evaluated in moderate to well differentiated and poorly differentiated carcinomas compared to matched normal tissue using TMA. In addition, the correlation between these epigenetic biomarkers and various clinicopathological factors, including, age, location, and stage of the disease were analyzed.

Seventy-seven and 34% of tumors were distal in Iranian and African American patients, respectively. In both populations, the percentage of methylation was >65% for SYNE1, MMP2, APC2, GPNMB, EVL, PTPRD, and STARD8, whereas methylation was <50% for LGR6, RET, CD109, and RNF. The difference in methylation between the two populations was statistically significant for CHD5, ICAM5 and GPNMB. Thirty-one percent AA tumors showed MSI-H, compared to 28% in Iranians.

**Conclusions/Significance:**

A significantly higher methylation rate was found for GPNMB, ICAM5, and CHD5 genes in AA patients compared to Iranians. These genes might play a role in the high incidence and aggressiveness of CRC in the AA population. The hypermethylation of the CAN genes can be considered as a marker of colon carcinogenesis.

## Introduction

Colon cancer (CRC) remains the most prevalent gastrointestinal cancer in the United States [1]. The incidence and mortality rate of CRC are higher in AA [2], [3]. A significant increase in CRC incidence with a predominant distal localization has also been reported in Iran over the last decade [4], [5].

One of the CRC pathways involves transcriptional silencing by hypermethylation of CpG islands, which is referred to as the methylator phenotype (CIMP^+^) [6] that mostly targets promoter regions of tumor suppressor genes (e.g., p16 and hMLH1 genes) [7], [8], [9]. It has recently been shown that genetic and epigenetic alterations of some candidate cancer genes (CAN genes) including; SYNE1, MMP2, GPNMB, APC2, EVL, PTPRD, CDH5, LGR6, STARD8, CD109, ICAM5, CHD5, RNF, and RET, are important in the progression of CRC [10], [11].

The functional characterization of these genes with regards to tumor progression has not been clarified completely. These genes could be divided into 5 classes: (1) tumor suppressors, including adenomatous polyposis coli tumor suppressor homolog 2 (APC2) and protein-tyrosine phosphatase receptor type-delta (PTPRD); (2) genes that encode receptors, including rearranged during transfection proto-oncogene (RET), leucine-rich repeat-containing G protein coupled receptor 6 (LGR6) and Ena/VASP like protein (EVL); (3) genes known to be involved in protein–protein and protein–DNA interactions, including STAR-related lipid transfer (START) domain–containing 8 (STARD8), ring finger protein (RNF182), CD109 antigen (CD109), glycoprotein NMB (GPNMB); (4) genes involved in metastasis and tumor growth, including intercellular adhesion molecule 5 (ICAM5), matrix metalloproteinase 2 (MMP2), and synaptic nuclear envelope protein 1 (SYNE1); and (5) genes whose expression is associated with changes in chromatin structure, such as chromodomain helicase DNA-binding protein 5 (CHD5).

These genes were chosen from many lists of potential cancer genes [11], [12] because recent studies showed that in non-AA CRC, their promoters are methylated and/or mutated [10]. This is, in fact, based on the comprehensive analysis by Sjoblom et al, where systematic sequencing of CRC tumors revealed the importance of these markers in CRC progression [11].

While most of these genes are novel, there is some functional data available in the literature. The receptors for glycoprotein hormones such as LGR6 are G protein–coupled 7-transmembrane receptors [13]. SYNE1 contains multiple spectrin repeats and a 60-amino acid C-terminal region homologous to the *Drosophila* protein Klarsicht. There are two mRNA isoforms, SYNE1A and SYNE1B, in skeletal and cardiac muscle [14], [15].

The metastatic potential of tumor cells has been found to correlate with the activity of MMP2 enzyme [16] that are functionally active on the surface of angiogenic blood vessels[16]. CD109 is a GPI-linked cell surface antigen expressed by CD34+ acute myeloid leukemia cell lines, T-cell lines, activated T lymphoblasts, endothelial cells, and activated platelets [17]. The RING finger motif is a specialized zinc finger domain including RNF182 and is found in many transcriptional regulatory proteins [18]. Mutations in the RET gene are associated with multiple endocrine neoplasia, type IIA and IIB [19].

Alteration of CHD5 expression is associated with changes in chromatin structure, through histones modification by acetylation and methylation [20]. It was noted that soluble ICAM5 level increased in the colony-stimulating factor of patients with acute encephalitis [21]. GPNMB is preferentially expressed in low-metastatic melanoma cell lines as glycoprotein [22]. In melanoma metastasis, there is an inverse relationship between the expression of GPNMB and calcyclin or thymosin-beta-10, two other potential markers for the progression of cutaneous melanoma. Two-thirds of highly metastatic melanomas expressing recombinant GPNMB showed slower subcutaneous tumor growth, whereas one-third showed reduced potential for spontaneous metastasis in nude mice [22] or iris pigment dispersion in DBA/2J mice [23]. APC2 is involved in a series of molecular signals initiated by the binding of Wnt protein to a frizzled family receptor on the surface of the target cell and ending with a change in cell state [24], [25]. APC2 protein interacts with a microtubule-associated protein, which effects beta-catenin-mediated growth signaling [24], [25]. The co-expression of the EVL protein along with alpha-II spectrin reinforces cell–to-cell interaction. The methylation of the EVL gene in all poorly differentiated tumors suggests that it is a factor in cell invasiveness [26]. STARD8 was identified as a tumor suppressor gene that inhibits cancer growth [27]. It is located on chromosome Xq13 and encodes DLC-3 (related to Rho GTPase). Transfection of human breast and prostate cancer cells with a DLC-3alpha expression vector inhibited cell proliferation, colony formation, and growth in soft agar [27].

In the present study, we analyzed samples from AA and Iranian patients for methylation of CAN genes' promoters. We hypothesized that CAN genes were inactivated through epigenetic silencing and may show distinct methylation profiles in different populations.

## Materials and Methods

### Ethics Statement

This study was approved by Howard University Institutional Review Board, and written informed consent was obtained.

### Study population, and tumor samples

A total of 102 CRC samples were used. Fifty-one sporadic CRC samples from Iranian patients, recruited at the hospitals of Shiraz University of Medical Sciences from 2003 to 2005 and 51 CRC samples from AA patients, recruited at Howard University Hospital, matched by sex, age (with ±5 years) and stage, were included in this study. All samples were evaluated and subjected to histological diagnosis by expert pathologists. Tissues were collected (with approval from all above sites' Institutional Review Boards and clinical data was obtained (including race, age, site of primary tumor, stage, and tumor differentiation). Family history of cancer was analyzed to exclude those pedigrees that met either the Amsterdam I or Amsterdam II criteria.

### Methylation-specific PCR

The promoter methylation status of the CAN genes was determined as described previously [28], [29], [30]. The sequences of primers used for amplification of the promoter regions of each of the CAN genes are listed in Table 1. The MS-PCRs were performed as previously described (Table 1) [10]. The MSP primers were designed using a software developed at the Johns Hopkins University (www.mspprimers.org) [10] based on the sequences for which the accession numbers and corresponding function are given in Table 2. The PCR conditions were as follow: hotstart Taq polymerase (Qiagen) used with initial activation and denaturation 95°C×15 min; 35 cycles [95°Cx45 sec; 60°C×45 sec; 72°C×1 min] followed by final extension 72°C×10 min. In vitro methylated DNA and unmethylated lymphocytes DNA were used as positive and negative controls, respectively. The annealing temperature was 56°C for APC2 and CD109 [10], respectively, while it was 60°C for all other genes (Table 1).

**Table 1:** Primers used in this study.

Gene	Sense sequence	Antisense sequence	Product size (bp)	Annealing Temperature (°C)	No. Cycles
APC2**Unmeth**	5′- tGGtAGtGttGttTGtttAGGtttGGAttG -3′	5′- ACCaaAAATCCCaaCCCaaaaTaaCCTCaaAaCa -3′			
APC2**Meth**	5′- GtCGttTGtttAGGttCGGAtC -3′	5′- GaCCCGaaaTaaCCTCGaAaCG -3′	98	**56**	35
CD109**Unmeth**	5′- GtAGtGGAtTGTAGtttAGGtAGAtGttGTtG -3′	5′- CaCaaCaaTaCACACaCAaAaaAaaTaaaCaaCa -3′			
CD109**Meth**	5′- GAtTGTAGtttAGGtAGACGtCGTC -3′	5′- CGaTaCACACGCAaAaaAaaTaaaCGaCG -3′	79	60	35
CHD5**Unmeth**	5′- GGGAGGAGtGtttGGGtTTTGtG -3′	5′- CaaCaaaCaAaaCaaCCTCaaCaAaAAaATaaCa -3′			
CHD5**Meth**	5′- GAGCGttCGGGtTTTGC -3′	5′- CGaCCTCGaCGAaAAaATaaCG -3′	119	60	35
EVL**Unmeth**	5′- GtGtGttTtTtttTtGAGGAtTtGGAGttGtttG -3′	5′- aCCaCCaaaaaATaaaaaaaCaaaaaaCaAaCCa -3′			
EVL**Meth**	5′- GAGGAtTCGGAGtCGttC -3′	5′- CCGAaaaATaaaaaaaCGaaaaaCGAaCCG -3′	119	60	35
GPNMB**Unmeth**	5′- AGGttTGAGAtGTGGGttGtGttttG -3′	5′- CCAAAAACaTAaaCaTTTTCCCaaaTCaCAaTCa-3′			
GPNMB**Meth**	5′- ACGTGGGtCGCGtttC -3′	5′- TAaaCGTTTTCCCGAaTCGCAaTCG -3′	88	60	35
ICAM5**Unmeth**	5′- tttAGttTTGtGTtttGGtTttGTGTTtTTtAttG -3′	5′- TCCTaaCAaAATaCCaaaATACaAaaAaAaTaCa -3′			
ICAM5**Meth**	5′- CGTttCGGtTtCGTGTTtTTtAtC -3′	5′- CTaaCAaAATaCCGAaATACGAaaAaAaTaCG -3′	116	60	35
LGR6**Unmeth**	5′- tGGGtAGGGGtAtGGttAGGtG -3′	5′- CCCTAaCTaCACaCACaTACCCaaaAaCTAAaCa -3′			
LGR6**Meth**	5′- GtAGGGGtACGGttAGGC -3′	5′- GCACGTACCCGAaAaCTAAaCG -3′	94	60	35
MMP2**Unmeth**	5′- GtGGttAtAtGtAttGAGttAGtGAtttttGGGtG -3′	5′- AaaAaACAaAaCaCCCTCAaaaaACCCaTaAaCa -3′			
MMP2**Meth**	5′- tAtCGAGttAGCGAttttCGGGC -3′	5′- CGCCCTCAaaaaACCCGTaAaCG -3′	96	60	35
RET**Unmeth**	5′- ttGGttttGttTGGtttAttttTGGAttGtttttG -3′	5′- CTaCaCaCCCTaCTTCaaTCaCaaaACTaAAaCa-3′			
RET**Meth**	5′- GGtttCGttTGGtttAttttTGGAtCGttttC -3′	5′- CTaCTTCGaTCGCGAaACTaAAaCG -3′	104	60	35
RNF182**Unmeth**	5′- GGtGGtTtAGtGttGTAGAGAtAAAGttGtttG -3′	5′- AaaaCCCaaaAaCCaCTCCaaCTaCaaCa -3′			
RNF182**Meth**	5′- tTtAGCGtCGTAGAGAtAAAGtCGttC -3′	5′- GCTCCGaCTaCGaCG -3′	109	60	35
STARD8**Unmeth**	5′- tAGGGAttGGGtTGGtTtTtGttGAGttttG -3′	5′- aTaaaaAaCTTCTAaaaCCaaCaaaaCTaTaCCa -3′			
STARD8**Meth**	5′- GGGtTGGtTtTCGtCGAGtttC -3′	5′- TTCTAaaaCCGaCGaaaCTaTaCCG -3′	90	60	35
SYNE1**Unmeth**	5′- GtGGtTGGGtTtttGtAGTttTGtAGAttGtG -3′	5′- CaaCTCTCTaCaCCCAaaCTCaaCa -3′			
SYNE1**Meth**	5′- GtTGGGtTttCGtAGTttTGtAGAtCGC -3′	5′- CTaCGCCCAaaCTCGaCG -3′	87	60	35
PTPRD**Unmeth**	5′- tGGtGGGGTttGtttAGGttGtG -3′	5′- ATaCTCCaAaCaCCCaCTaaaaAaAaAAaCaaCa -3′			
PTPRD**Meth**	5′- GGGGTtCGtttAGGtCGC -3′	5′- CGCCCGCTaaaaAaAaAAaCGaCG -3′	120	60	35

**Table 2:** CAN genes, their functions andcorresponding Accession number.

Gene	Gene Name	Gene Function	References	Accession Number
APC2	Adenomatosis polyposis coli 2	Wnt transduction pathway	[25], [26]	NM_005883.2
CD109	CD109 molecule	Expressed in CD34+ acute myeloid leukemia and other blood cell lines	[17]	NM_133493.2
CHD5	Chromodomain helicase DNA binding protein 5	Intervenes in chromatin modification through histones acetylation and methylation	[21]	NM_015557.1
EVL	Enah/Vasp-like	Strenghtens cell-to-cell interaction	[27]	NM_016337.2
GPNMB	Glycoprotein (transmembrane) nmb	Expressed in low metastatic melanomas cell lines	[23]	NM_001005340.1
ICAM5	Intercellular adhesion molecule 5, telencephalin	Highly expressed in the colony-stimulating factor of patients with acute encephalitis	[22]	NM_003259.2
LGR6	Leucin-rich repeat-containing G protein-coupled receptor 6	Receptor for glycoproteins Hormones	[13]	NM_021636.2
MMP2	Matric metallopeptidase 2	Active on angiogenic blood vessels, metastasis	[16]	NM_004530.2
PTPRD	Protein tyrosin phosphatase, receptor-type, D	TSG, involved in a wide range of common human cancers	[51]	NM_130391.2
RET	Ret proto-oncogene	Associated with multiple endocirine neoplasias type IIA and IIB	[19]	NM_020975.4
RNF182	Ring finger protein 182	Found to many transcriptional regulatory proteins	[18]	NM_152737.2
STARD8	START domain contain 8	TSG, inhibits cancer growth	[27], [52]	NM_014725.2
SYNE1	Spectrin repeat containing, nuclear envelope 1	Expressed in skeletal and cardiac muscles	[14], [15]	NM_182961.1

### DNA isolation and MSI analysis

Archived and fresh tumor blocks were cut into 5-µm sections on Superfrost slides (Fisher Scientific, Pittsburgh, PA). The tumor and normal areas were diagnosed by a pathologist using the H&E matched slide and microdissected to pinpoint the tumor and normal areas from at least two slides. DNA extraction and MSI (five microsatellite markers [31] (BAT25, BAT26, D17S250, D5S346, and D2S123) were done according to our previous studies [28], [29], [30]. Tumors with instability at only one of the markers were labeled MSI-L, those with instability in two or more markers were labeled MSI-H, and those with no instability were labeled MSS. Due to unclear characteristics of MSI-L, we combined MSS and MSI-L into one group (non-MSI-H).

### Tissue Microarrays and Immunohiostochemical Analysis

Tissue microarrays (TMAs) were constructed using a Beecher Instruments MTA-1 tissue arrayer. Each TMA contained tissue from normal and tumor areas, based on a published protocol [32]. Duplicate tumor samples were taken from each tissue block. In total, 116 cases were analyzed for CHD5 expression; moderate to well differentiated (55 cases), and poorly differentiated (4 cases) carcinomas with matched adjacent normal tissues (57 cases) were available for control comparisons. A retrospective analysis for outcome assessment was based on detailed clinicopathological information linked to the TMA specimens. TMA obtained from paraffin-embedded blocks was used for the immunohistochemistry experiments. Sections (5 µm) were mounted on charged glass slides, deparaffinized with xylene for 2×10 min and rehydrated using a graded ethanol series. Antigen retrieval was performed by placing the samples in a microwave oven for 12 min, with occasional interruption to avoid tissue degradation by excessive heat. The slides were then treated with hydrogen peroxide, followed by incubation with the primary and secondary antibodies, a streptavidin-biotin complex, an amplification reagent, streptavidin-peroxidase and substrate-chromogen solution using the Envision system according to the manufacturers' protocol (DAKO). The samples were then counterstained with hematoxylin, rinsed with ethanol, dried and visualized by light microscopy. Tissue samples to which no primary antibody had been added were used as negative controls. All immunohistochemistry reagents were purchased from DAKO (Carpinteria, CA). The CHD5 antibody (CHD5 clone H-185, 1/10 dilution) was purchased from Santa Cruze (San Diego, CA). The slides were read by two pathologists (E.L; R.G.) and the percentage of the cytoplasmic staining was recorded.

### Histopathological analysis

Independent pathologists evaluated specific histopathological characteristics. Grading of tumors was achieved by staining with Hematoxylin-Eosin (H&E). Tumors were classified as proximal or distal (to the splenic flexure). The TNM system of the International Union against cancer was used for tumor staging.

### Statistical analysis

Age of patients was a continuous variable, while race, gender, location, differentiation, stage, MSI, and CAN genes methylation were categorical variables. The distribution of categorical variables were shown by frequency table, and for age by computing mean (SD). Associations between methylation of loci with age, race, gender, differentiation, MSI, stage and tumor location were evaluated using a chi square test. The age difference between two groups was tested by the Student's *t* test. All analysis were performed using *SPSS 15.0* software (Chicago, IL).

## Results

### Clinicopathological characteristics of patients

We analyzed 102 samples (38 females and 64 males) from Iranian and AA patients (Table 3). The mean age (SD) for carcinoma in AA was 61.5 (12) years and 60 (13) years in Iranians. There was no significant difference in sex or age between the two analyzed populations. A total of 57% and 24% of tumors were proximal in the AA and Iranian patients, respectively. A higher incidence of distal tumors was present in Iranians in comparison to the AA (Table 3). Most tumors were at advanced stages with 57% at stage II in Iranians, and 52% at stage III+IV in AA (Table 3). The majority of tumors (85%) were found to be moderately differentiated in AA while Iranian tumors were mostly well differentiated (53%).

**Table 3:** Clinical and demographical characteristics of CRC in the two populations.

	African Americans	Iranians	P
Number of patients	51	51	
Mean (SD) age	61.5 (12)	60 (13)	0.5
Gender	N (%)	N (%)	
Female	19 (37)	19 (37)	
Male	32 (63)	32 (63)	1.0
Site			
Distal	22 (43)	39 (77)	
Proximal	29 (57)	12 (24)	0.001
Age			
<60	24 (47)	29 (57)	
≥60	27 (53)	22 (43)	0.002
Differentiation			
Poor	2 (4)	2 (4)	
Moderate	39 (85)	22 (43)	
Well	5 (11)	27 (53)	0.0001
Stage			
0,1	9 (21)	8 (15)	
2	12 (27)	29 (57)	
3,4	23 (52)	14 (28)	0.01
MSI			
High	10 (20)	14 (28)	
Non- High	40 (80)	37 (72)	0.2

### SYNE1 and RNF182 gene methylation profiles

The SYNE1 promoter was found to be methylated in all 102 analyzed samples (Table 4). In addition, its methylation does not seem to be specifically associated with any of the clinicopathological parameters considered in this study and point to its importance in colon tumorigenesis as a tumor suppressor gene. In contrast, the RNF182 gene promoter was unmethylated in all analyzed samples (Table 4). This finding will put in question its status as a candidate for methylation in colon cancer carcinogenesis.

**Table 4:** Methylation (%) comparison between two populations.

Gene	Iranians	AA	P-value
APC2	48 (94)	49 (96)	0.6
SYNE1	51 (100)	51 (100)	1
GPNMB	45 (89)	50 (100)	0.03
EVL	41 (79)	35 (71)	0.4
MMP2	51 (100)	48 (94)	0.2
CD109	16 (32)	15 (30)	0.8
CHD5	25 (47)	38 (78)	0.002
RNF182	0	0	1
LGR6	16 (31)	25 (49)	0.8
PTPRD	38 (76)	38 (76)	1
STARD8	33 (65)	38 (75)	0.3
RET	19 (37)	21 (41)	0.7
ICAM5	4 (7.5)	20 (40)	0.0001

### Gender and CAN gene methylation

Nine out of 13 genes showed gender-independent levels of methylation (Table 5). The RET gene displayed a higher level of methylation in males (45%) than in females (29%). This difference, however, was not found to be statistically significant. The APC2, PTPRD and STRAD8 genes were found to have significantly different methylation profiles in the two genders, with APC2 being hypermethylated in males (98% vs. 90%); PTPRD and STARD8 were hypermethylated in females (90% vs. 67%) and (84% vs. 61%), respectively.

**Table 5:** Distribution of the CAN genes methylation (%) by demographic and clinical characteristics of CRC in the AA and Iranian populations.

N = 102		APC2	GPNMB	EVL	MMP2	CD109	CHD5	LGR6	PTPRD	STARD8	RET	ICAM5
Gender	Male	98*	95	72	97	34	64	37	67**	61*	45	23
	Female	90*	92	81	97	24	60	42	90**	84*	29	24
Age	<60	96	94	74	98	25	66	38	77	72	47	19
	≥60	94	94	77	96	37	58	43	71	67	31	29
Location	Distal	95	93	74	98	21*	57	33	72	69	39	16*
	proximal	95	95	78	95	43*	71	51	78	71	39	34*
Differentiation	Poor	100	67	100	75*	0	67	50	75	50	0	0**
	Moderate	97	97	77	98*	31	66	38	72	69	39	34**
	Well	91	91	75	97*	34	50	38	78	69	38	6**
Stage	0,1	82*	94	65	94	24	82	71**	77	71	35	24
	2	98*	93	78	100	27	49	24**	71	66	42	15
	3,4	97*	94	75	95	35	61	43**	78	70	35	32
MSI	High	100	91	83	100	33	61	38	92*	75	42	29
	Non-MSI	94	95	72	96	30	62	40	70*	68	38	21

SYNE1 and REN were fully methylated and unmethylated, respectively for all samples tested.

* p<0.05 **p<0.01.

### Age and CAN gene methylation

None of the 13 genes analyzed showed any age-dependent methylation profile, although ICAM5 and CD109 showed non-statistically significant differences (Table 5). This finding is consistent with the way the genes were chosen and supports the idea that most of them are targeted by methylation in a carcinogenic process.

### Tumor location and CAN gene methylation

Ten of the analyzed genes have similar methylation levels, regardless of the tumor location (Table 5). However, CD109, LGR6, and ICAM5 displayed higher methylation levels in proximal tumors than in distal ones. Methylation frequency of CD109 was 43% in proximal tumors vs. 21% in distal tumors. These numbers for ICAM5 were 34% in proximal and 16% in distal tumors, respectively (p<0.05). For the LGR6 gene, the difference (51% vs. 33%) was not statistically significant.

### Tumor differentiation and CAN gene methylation

While 8 genes displayed different methylation profiles at different levels of differentiation, only two showed statistically significant differences (ICAM5 and MMP2). However, there was no correspondence with tumor progression toward poor differentiation (Table 5). Only the APC2 and EVL genes displayed higher methylation in the normal progression of a tumor from well to moderate to poor differentiation, with the APC2 gene showing 91%, 97%, and 100% and the EVL gene showing 75%, 77%, and 100%, respectively. These findings might underscore the role of these genes in the tumor differentiation process.

### Tumor stage and CAN gene methylation profile

Eight genes showed different methylation profiles at different tumor stages, with the LGR6 and APC2 genes displaying statistically significant differences with higher methylation at stage 1 and lower methylation at advanced stages in the case of LGR6 and lower methylation at stage 1 and higher methylation at advanced stages in the case of APC2 (Table 5). The only gene that showed a higher methylation at more advanced tumor stages was CD109, which was methylated at a rate of 56%, 76%, and 86% at stages I, II and (III+IV), respectively.

### MSI and CAN gene methylation

The MSI rate was 28% for Iranians and 31% for AA (Table 3), respectively. Twelve of the 13 tested genes showed no differences in methylation levels between MSI-H and non MSI-H tumors in both populations. An exception could be made for the PTPRD gene with a statistically significant association with MSI-H (P<0.05) in both populations (Table 5). Therefore, there is a possibility that the methylation of PTPRD is linked to the MSI-H phenotype.

### Population to Population comparison

**Figure 1:**
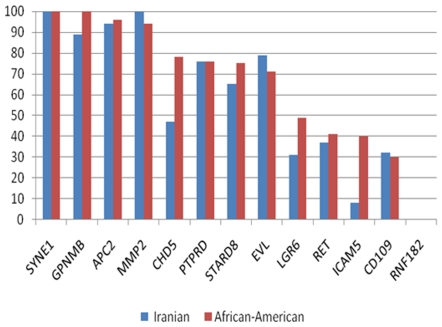
Graphical presentation of methylation frequencies (%) of the CAN genes in the studied populations.

The methylation profiles of at least 9 genes in the two analyzed populations was similar without significant differences (Table 4 and figure 1): APC2, SYNE1, EVL, MMP2, CD109, RNF182, PTPRD, STARD, and RET. For the LGR6 gene, there was a substantial difference, although statistically insignificant, in methylation levels with 31% vs. 49% in Iranians and AA, respectively. For three genes, namely GPNMB, CHD5, and ICAM5, there were statistically significant differences in the methylation level, with AA displaying higher methylation levels than Iranians, 100%, 78%, and 40% vs. 89%, 47%, and 7.5% for the three genes, respectively (Table 4)

### CHD5 expression by IHC, Differentiation and Tumor Stage

**Figure 2:**
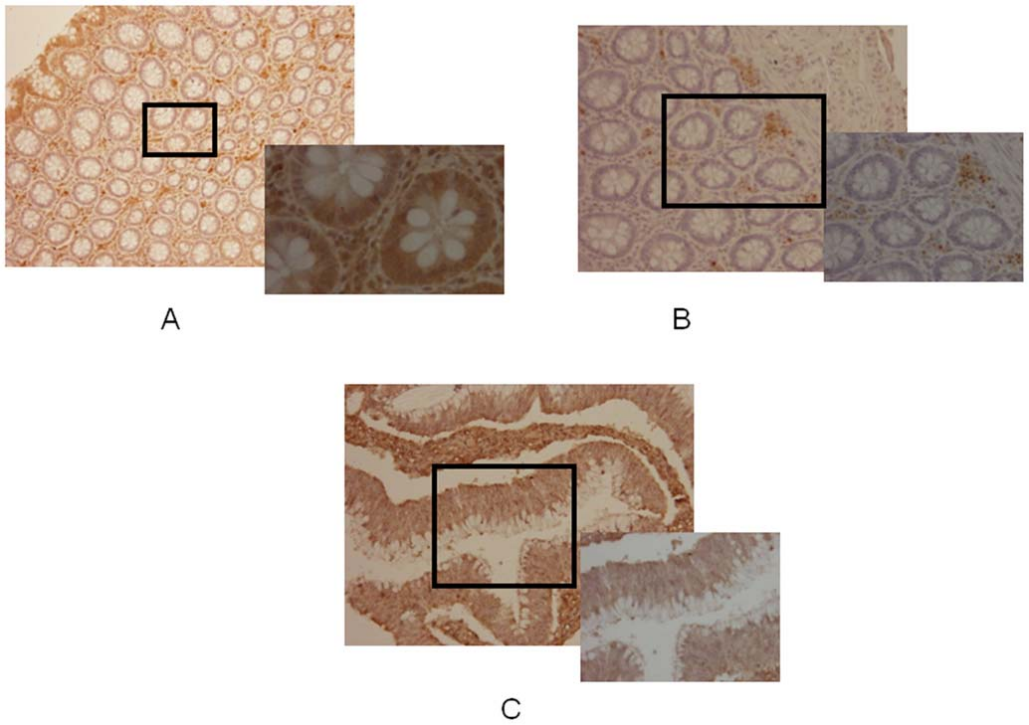
Immunohistochemical staining of CHD5 in human tissue microarray (A, B, C). (A) Positive CHD5 staining evident in all of the normal glands in biopsy specimens from normal colon biopsies (B) in normal patients lack of brown color indicates absence of cytoplamic staining for CHD5 in absence of primary antibody, (C) in CRC patients (>52%) of the cases showed absence of cytoplasmic staining for CHD5 in the malignant epithelial cells.

Since one of our laboratory's main focuses is to tackle the issue of the high incidence of CRC in AA and, based on the fact that CHD5 promoter is highly methylated in this population and seems to be involved in early stages of carcinogenesis as a chromatin modifier, we analyzed its expression by IHC to validate the methylation results. Among 59 CRC cases available for analysis, the number of subjects with stage I, II, III, and IV were 14 (24.5%), 20 (35.1%), 21 (36.8), 2 (3.5%), and 2 (missing) respectively. In general, there were no statistically significant differences for age, sex, anatomic location, CHD5 expression with tumor stage (data not shown). Expression of CHD5 (Fig. 2C) was lost in 80% of AA patients and 52% in Iranian patients with stage II and III disease, respectively. However, the difference was not statistically significant. The loss of CHD5 expression was consistent with CHD5 methylation in CRC. The cytoplasmic expression of CHD5 was present in normal colon epithelial cells (Fig. 2A) compared to the negative control without the primary antibody (Fig. 2B), indicating the specificity of the antibody.

## Discussion

Epigenetic analysis of tumor cells plays a major role in the understanding of carcinogenic processes and targeted therapies [7]. Sjoblom et al. have sequenced thousands of genes in 11 primary breast and colon tumors and concluded that every single tumor has an average of 14 genetic alterations [11]. A subsequent epigenetic and mutation study [10] led to the identification of silenced promoters, 13 of which correspond to the CAN genes analyzed in this study [11]. We decided to investigate the impact of these 13 genes in CRC using two different sample populations. Our choice of the 13 genes was based on the fact that they were established from a high throughput technology and a comprehensive study involving both cell lines and clinical white colon samples that were validated using DNA from normal and cancer tissue [10], [11], [33]. Here, we analyzed the methylation profile of these 13 genes in AA and Iranian CRC, the MSI status and the expression by IHC of CHD5, a gene suspected to be involved in early carcinogenic processes.

Most of the analyzed genes were highly methylated with different levels of methylation from one gene to another and one population to the other. SYNE1, a synaptic nuclear envelope encoding protein, was methylated in all analyzed samples while the RNF182 promoter, encoding a ring finger protein, was methylated in none. No known function for the RNF182 gene is available to date. SYNE1 protein was shown to be involved in the process of cytokinesis [34] where this protein and KIF3B protein facilitate the accumulation of membrane vesicles at the spindle midbody.

The methylation profile of the analyzed genes was shown to be independent of age (Table 5). While there is a general methylating process that is age dependent and is not gene/disease specific, the results obtained with the analyzed genes reflect the relevance of these genes in the process of carcinogenic-dependent methylation. These findings are strengthened by the fact that many of the patients involved in this study, especially Iranians, are relatively young (40 years).

At least four genes showed a level of methylation that depends on the patient gender. A higher level of methylation in male patients was found for APC2 and RET, while a higher level of methylation in female patients was displayed for PTPRD and STARD8 genes (Table 5). At least four genes (CD109, CHD5, LGR6, and ICAM5) displayed a different level of methylation, depending on the tumor location. These genes showed a lower level of methylation in the distal colon. This finding is in agreement with the presence of a descending methylation gradient from the proximal to the distal colon. A higher level of methylation from well to poorly differentiated tumors was observed only for the EVL gene promoter. Bournier et al. have shown that the co-expression of the EVL protein along with alpha-II spectrin reinforces the cell-to-cell interaction [26]. The methylation of the EVL gene in all poorly differentiated tumors and more than 75% of well and moderately differentiated ones increases their invasiveness. This finding is strengthened by the fact that this gene methylation increases also in advanced stage tumors where only 65% of stage-I tumors were methylated compared to 75% at stage IV. An apparently stage-dependent methylation was observed for LGR6, which encodes a Leucine-rich repeat-containing G-protein- coupled receptor, and is thus involved in cell proliferation [13]. However, this decrease in methylation status from stage I to stage IV (71% to 43%) cannot be explained in light of this gene function as a proliferation promoter. APC2 stage-dependent methylation from stage I to stage IV (82% to 97%) was also observed and this may be consistent with the tumor suppressor activity of APC2 gene in CRC and its link to the Wnt pathway.

The multivariate analysis for the effect of confounders (site, differentiation and stage) is different among the two populations (Table 3). To be confounders these variables need to be correlated with methylation, too. As shown in table 5, ICAM5 is correlated with differentiation and site while GPNMB and CHD5 are not related to any of these variables. Based on these findings, site and differentiation may have a confounder role for ICAM5. To be consistent and inclusive in statistical analysis, we developed three logistic regression models using forward selection with each gene as dependent factor and site, population (Iranian vs. AA), stage, and differentiation as independent factors. For all three models AA remains the significant factor for methylation.

The methylation profile for all but one PTPRD gene was similar in both MSI-H and non-MSI-H tumors, confirming an already-established dissociation between the CpG island methylator phenotype (CIMP) and the microsatellite instability phenotype in colon cancer tumors. The PTPRD gene encodes a protein that is a member of the protein tyrosine phosphatase family, signaling molecules that regulate a variety of cellular processes including cell growth, differentiation, mitotic cycle, and oncogenic transformation. Mori et al. (2004) have already shown that PTPR type O is highly methylated in MSI-H tumors, strengthening our finding [35].

A population-to-population comparison reveals a different methylation profile between Iranians and AA for: GPNMB (89 vs. 100%), CHD5 (47 vs. 78%), LGR6 (31 vs. 49%), and ICAM5 (7.5 vs. 40%) with at least 3 statistically significant differences (GPNMB, CHD5 and ICAM5). GPNMB, a type-I transmembrane glycoprotein, shows expression in the lowly metastatic human melanoma cell lines but does not show expression in the highly metastatic cell lines [36]. This gene' product may be involved in growth delay and reduction of metastatic potential. Therefore, the higher methylation level of GPNMB in AA might partly account for the high aggressiveness and fast progression of colon tumors in AA. This finding is also reinforced by the fact that another gene involved in metastasis, ICAM5, is highly methylated in AA when compared to Iranians. ICAM5 encodes a type I transmembrane glycoprotein that is a member of the intercellular adhesion molecule (ICAM) family. High methylation level of ICAM5 decreases the cell-to-cell adhesion in the corresponding tumor cells, increasing their invasive potential. This finding is consistent with the GPNMB results leading to cumulative effects that increase the invasiveness and metastatic potential. Unlike GPNMB and ICAM5, CHD-5 (chromodomain helicase DNA binding protein 5) seems to be involved in early tumorigenic processes at the chromatin remodeling level and controls events, such as proliferation, apoptosis, and senescence, via the p19(Arf)/p53 pathway [37]. The methylation level of this gene in AA (78% vs. 47% in Iranians) might reflect the high level of incidence of colon cancer in AA. Indeed, chromatin modification affects the expression profiles of many genes at once and impacts the quick progression of the tumor. Our recent publications have shown that AA colon tumors display an aberrant global histone (H3, and H4) acetylation and HDAC2 expression [38]. The hypermethylation of those genes that showed similarities between the two populations may be an early silencing marker for CRC initiation.

Based on the obtained results and known characteristics of AA CRC, the CAN genes methylation results support the highly methylated CHD5 and ICAM5 in the AA tumors, pointing to a prominent role of CHD5 and ICAM5. There was a consistent result between CHD5 methylation and lack of CHD5 protein expression using IHC (Fig. 2). In addition, the expression and functional analysis of these genes will be an important perspective of this work that we are planning to address in future. Recently CHD5 has been referred to as a tumor suppressor gene, which supports our claim for epigenetic silencing [39] and its IHC expression analysis. The methylation of CHD5 is a participating factor in the higher incidence of CRC in AA along with other markers (genetic and epigenetic). Differences in dietary, environmental, and molecular genetic factors may also play a role [2], [40] [41], [42]. Racial disparities have been observed in lipoxygenase polymorphisms [43], microsatellite instability [44], folate metabolic gene polymorphisms [45], and vitamin D receptor haplotypes [39], [46].

The CAN genes could be referred to as CIMP markers since there is no agreed upon standard CIMP list and different laboratories have different CIMP genes list [12], [47], [48], [49], [50].

In conclusion, our study confirms the hypermethylation of cancer candidate genes as biomarkers and a higher methylation profile of GPNMB, ICAM5, and CHD5 genes in AA was observed. Therefore, this may explain to certain extent the high incidence and aggressiveness of CRC in AA. For a global view of epigenetic processes in colon tumorigenesis in these groups of patients, a thorough analysis of both populations' tumors might need to be done on established cell lines using agents targeting both whole-genome methylation and/or chromatin modification inhibitors followed by differential microarray expression studies.
